# Clinical Predictors of Intracranial Pathology in Emergency Department Patients with Non-traumatic Headache and No Neurological Deficits: Prospective Study

**DOI:** 10.5811/westjem.48670

**Published:** 2026-01-27

**Authors:** Mustafa Serinken, Cenker Eken, Faruk Güngör, Ömer Akdağ, Veli Citisli

**Affiliations:** * Denipollife Hospital, Department of Emergency Medicine, Denizli, Türkiye; † ASV Yaşam Hospital, Department of Emergency Medicine, Antalya, Türkiye; ‡ Isparta State Hospital, Department of Emergency Medicine, Isparta, Türkiye; § Pamukkale University, Department of Neurosurgery, Denizli, Türkiye

## Abstract

**Introduction:**

Non-traumatic headache is a common emergency department (ED) presentation, yet identifying intracranial causes remains challenging in the absence of neurological deficits. In this study we aimed to evaluate the incidence and predictive ability of clinical red flag signs and symptoms for intracranial pathology.

**Methods:**

We conducted a prospective, multicenter, cross-sectional study across six academic EDs with residency programs in Türkiye. We enrolled consecutive adult patients with non-traumatic headache and no neurological deficits who had cranial computed tomography (CT) at the discretion of the treating attending physician. Exclusion criteria were recent trauma, pregnancy, fever, hematologic conditions, and known intracranial pathology. We recorded clinical features using standardized forms. The primary outcome was the presence of intracranial pathology confirmed by CT or subsequent diagnosis within a one-month follow-up.

**Results:**

Of 1,522 patients, 57 (3.7%, 95% CI, 2.8–4.8) had intracranial pathology; 104 (6.8%) patients could not be reached during the one-month follow-up. The most common diagnoses were subarachnoid hemorrhage (SAH) (n = 20, 35.1%); ischemic stroke (n = 16, 28.1%); subdural hemorrhage (n = 6, 10.5%); and sinus vein thrombosis (n = 6, 10.5%). Both univariate and multivariate analyses identified that headache aggravated by physical activity (OR 5.98; 95% CI, 2.3–15.2) and age > 50 years (OR 3; 95% CI, 1.65–5.5) independently predicted the cause of the headache. For SAH, headache exacerbated by physical activity (OR 18.6; 95% CI, 5.6–62.3), and syncope (OR 5.7; 95% CI, 1.4–24.3) were independent risk factors. Notably, “sudden onset” and “worst headache ever” were not significant predictors of intracranial pathology in this cohort. The prevalence of sudden-onset headache (45%, n = 9, vs 50.3%, n = 753; *P* = .64) and “worst headache ever” (55%, n = 11, vs 59.4%, n = 890; *P* = .69) did not differ significantly between patients with and those without SAH. The odds ratios from the multivariable analyses for sudden onset (OR 1.13, 95% CI, 0.4–3.0) and “worst headache ever” (OR 1.38, 95% CI, 0.47–4.0) were not statistically significant for SAH.

**Conclusion:**

In ED patients presenting with non-traumatic headache and no focal neurological deficits, headache aggravated by physical activity is a significant indicator for any intracranial pathology causing headache and, specifically, for subarachnoid hemorrhage. While age > 50 years was associated with intracranial pathology causing headache, syncope was specifically linked to subarachnoid hemorrhage. These findings may help refine clinical decision-making for neuroimaging in this patient population.

## INTRODUCTION

Headache is the fifth most common reason for presentation to the emergency department (ED), while migraine headache ranks as the sixth leading cause of disability globally, according to the Global Burden of Disease Report.^1^,^2^ The International Headache Society classifies headaches into two categories: primary and secondary.^3^ Primary headaches typically require symptomatic management, whereas secondary headaches necessitate further diagnostic evaluation, including imaging modalities such as computed tomography (CT) and procedures such as lumbar puncture (LP) in the ED setting.

Although not all secondary headache disorders demand immediate neuroimaging, certain life-threatening conditions—such as subarachnoid hemorrhage (SAH)—require urgent investigation. Most headache presentations in the ED are due to primary headache disorders, and the incidence of serious pathological diagnoses is relatively low, estimated at 2%. Despite this, 14% of patients presenting with headache undergo imaging, predominantly CT, yet the diagnostic yield remains limited, with only 5.5% of these studies revealing clinically significant findings.^4^

Patients presenting with primary headaches similar to their previous episodes, or those with neurological deficits, generally do not pose a diagnostic dilemma when deciding to perform a CT. However, patients who do not fall into these categories still present a challenge when considering CT imaging to identify potential intracranial cause. Moreover, CT is not without risk, as it exposes patients to ionizing radiation associated with an increased risk of cancer. In 2023, 93 million CTs were performed in the United States, which was projected to result in approximately 103,000 future cancer cases, according to a recent study published in the *Journal of the American Medical Association*.^5^ Nevertheless, selecting patients for CT imaging more judiciously could help prevent unnecessary exposure to radiation-related risks and reduce the associated economic burden.

In this study we aimed to identify clinical red flags indicative of intracranial causes in patients presenting with non-traumatic headache in the absence of neurological deficits, thereby addressing the diagnostic challenge of determining the necessity for CT.

## METHODS

### Study Setting

We conducted this prospective, multicenter, cross-sectional study within an 18-month period in four EDs of six tertiary-care hospitals in Türkiye. Each ED had an annual patient volume of 50,000, and the remaining two had 180,000. Ethical approval was obtained from the relevant institutional review boards prior to study initiation.

**Population Health Research Capsule**

What do we already know about this issue?

*Identifying intracranial causes of non-traumatic headache in patients without neurological deficit often leads to unnecessary computed tomography (CT)*.

What was the research question?

*What clinical red flags predict intracranial pathology in patients presenting with non-traumatic headache and no neurological deficits?*

What was the major finding of the study?

*Headache aggravated by physical activity is related to an intracranial cause (OR, 5.98; 95% CI, 2.3–15.2) and subarachnoid hemorrhage (OR 18.6; 95% CI, 5.6–62.3)*.

How does this improve population health?

*Awareness of headache aggravated by physical activity as a predictor of intracranial pathology in patients with non-traumatic headache could lessen unnecessary CT*.

### Selection of Participants

Patients presenting with non-traumatic headache and no neurological deficits who were deemed eligible for cranial CT due to a suspected intracranial cause were prospectively included in the study. In this context, secondary headache was operationally defined as a headache attributable to an intracranial cause, given its more urgent and critical clinical implications compared to extracranial causes such as sinusitis or glaucoma. Furthermore, since the study patients had to be without neurological deficits, all demonstrated normal mental status and normal findings on neurological examination. The neurological examination included assessment of mental status, lateralizing motor or sensory deficits, speech abnormalities, cranial nerve function, and cerebellar function. All patients presented to the ED were seen by residents under the supervision of an attending emergency physician. The decision to perform a CT was made by the attending physician before the resident ordered the study based on clinical judgment and adherence to inclusion and exclusion criteria, reflecting pragmatic, real-world ED practice. However, the study was not limited to a single diagnostic tool; the use of additional diagnostic methods was left to the discretion of the emergency physician. Patient recruitment occurred continuously, 24 hours a day, seven days a week.

### Exclusion Criteria

Exclusion criteria included the following: recent head trauma within the prior week; < 18 years of age; presence of neurological deficits; pregnancy; fever; a known diagnosis of primary brain tumor or metastatic brain lesions; and hematologic conditions such as aplastic anemia, lymphoma, or idiopathic thrombocytopenic purpura. Patients with a history of recent neurosurgery or hydrocephalus, as well as those who declined to provide informed consent, were also excluded. Additionally, due to a planned secondary analysis exploring the association between D-dimer levels and intracranial causes, we excluded from participation individuals with a history of deep vein thrombosis or pulmonary embolism.

### Data Collection

Data were collected by emergency medicine residents using a structured study form. This form captured patients’ demographic characteristics, confirmation of inclusion and exclusion criteria, and specific clinical features of the headache, including sudden onset, history of similar prior episodes, whether the current headache was the most severe ever experienced, associated symptoms such as vomiting or syncope, and response to analgesic treatment. Analgesic use was documented; however, the choice of medication and dosing regimen were not standardized and left to the discretion of the attending physician.

Radiologists interpreted all cranial CT in the respective institutions’ radiology departments. The emergency physicians ordered the CT without contrast, which has been shown to be cost-saving in patients presented to the ED with acute non-traumatic symptoms referable to the brain.^6^ The decision to perform contrast-enhanced CT was made by radiologists based on clinical findings, non-contrast CT results, and differential diagnoses such as tumors or venous thrombosis.

### Primary Outcome

The primary outcome of the study was the identification of intracranial causes of headache, including intracranial hemorrhage, cerebral venous thrombosis, brain tumors, and acute ischemic stroke. Extracranial etiologies such as sinusitis or mastoiditis—although occasionally visualized on CT—were not considered primary outcomes due to their lesser clinical urgency. To ensure comprehensive outcome assessment, a follow-up telephone interview was conducted one month after the initial ED visit to ascertain whether any alternative or delayed diagnosis had been established.

### Statistical Analysis

We analyzed study data using SPSS v23.0 (SPSS Statistics, IBM Corp, Armonk, NY)and MedCalc for Windows, v23.2.6 (MedCalc Software, Ostend, Belgium). The numeric data were expressed as mean ± standard deviation and the frequency data as rates. We performed a comparison of two independent groups with categorical variables by Pearson chi-square test and Fisher exact test. Logistic regression analysis was performed to establish the independent risk factors in suggesting intracranial causes of headache in the study patients. The hypothesis was constructed as two-tailed, and an alpha critical value of .05 was accepted as significant.

## RESULTS

**Figure 1:**
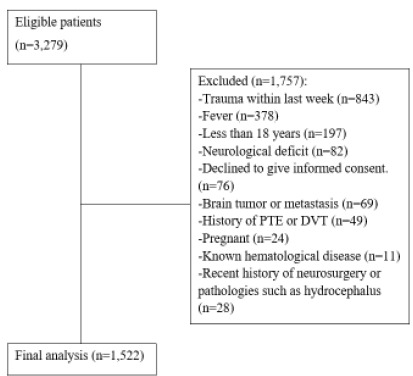
Patient flow chart in study of predictors of intracranial hemorrhage. *PTE*, pulmonary thromboendarterectomy; *DVT*, deep vein thrombosis.

Of 3,279 patients eligible for the study, we excluded 1,757 for various reasons. A total of 1,522 patients were included in the final analysis (Figure 1). Moreover, 104 (6.8%) patients could not be reached during the one-month telephone follow-up. The mean age of the study patients was 47.6±16.8, and 643 (42.2%) were male.

Of all patients included in the final analysis, 762 (50.1%) reported sudden-onset headache pain, 545 (35.9%) reported similar previous headaches, 92 patients (6.1%) reported headache with syncope, 71 (4.7%) with aggravation with physical activity, and 276 (18.1%) had an analgesic response to headache (Table 1). Fifty-seven patients (3.7%) were diagnosed with an intracranial pathology. The most prevalent intracranial causes among the study patients were as follows: 20 (35.1%) had SAH; 16 (28.1%) had ischemic stroke; six (10.5%) had subdural hemorrhage; six (10.5%) had sinus venous thrombosis; and five patients (8.8%) had a brain mass (Table 1).

No feature related to the patient’s present history was significant in predicting an intracranial cause with the exception of headache aggravated by physical activity and age > 50 years . More patients with an intracranial cause were > 50 years of age (68.4%, n = 39 vs 43.8%, n = 641; *P* = <.001) and stated their headache intensified by physical activity compared to patients without an intracranial cause (12.3%, n = 7 vs 4.4%, n = 64; *P* = .01) (Table 2).

Sudden onset of headache pain (28.1%, n = 16 vs 51.1%, n=746; *P* = <.001) and worst headache ever (42.1%, n = 24 vs 60%, n = 877; *P* = <.001) were significantly more prevalent in patients without intracranial pathology. Multivariate analysis also confirmed that age > 50 years (OR 3, 95% CI, 1.65–5.5) and headache intensified by physical activity (OR 5.98, 95% CI, 2.3–15.2) were independent risk factors in suggesting an intracranial cause.

Similar to any intracranial pathology, no identifying feature emerged in patients with SAH, except for headache aggravated by physical activity (25%, n = 5 vs 4.4%, n = 66; *P* = < .001). However, syncope was more prevalent in patients with SAH along with a borderline *P*-value that was not significant (15%, n = 3 vs 5.9%, n = 89; *P* = .09). The prevalence of sudden-onset (45%, n = 9 vs 50.3%, n = 753; *P* = .64) and “worst headache ever” (55%, n = 11 vs 59.4%, n = 890; *P* = .69) did not differ significantly between patients with SAH and those without SAH (Table 3).

Multivariate analysis confirmed the results of the univariate analysis that headache aggravated by physical activity (OR, 18.6, 95% CI, 5.6–62.3) is an independent risk factor in suggesting SAH. However, syncope (OR 5.7, 95% CI, 1.4–24.3) is also established as an independent variable as well as aggravation of headache by physical activity in multivariate analysis. Of note, the odds ratios from the multivariable analyses for sudden-onset headache (OR 1.13, 95% CI, 0.4–3.0) and “worst headache ever” (OR 1.38, 95% CI, 0.47–4.0) were not statistically significant for SAH.

## DISCUSSION

In the present study, no specific historical feature was found to be significantly more prevalent in patients with an intracranial cause of headache compared to the control group, except for headache aggravated by physical activity and age > 50 years, among those presenting with non-traumatic headache and no neurological deficits. There is limited evidence regarding red-flag indicators in the specific patient population assessed in this study—namely, individuals presenting with non-traumatic headache and no neurological deficits. Most prior studies have included patients with neurological abnormalities, which may have confounded the identification of more subtle clinical predictors. A small study by Muñoz-Cerón et al investigated patients > 18 years of age presenting with non-traumatic headache and found that age > 50 was associated with an increased risk of intracranial cause. However, no significant association was observed for sudden-onset headache or headache occurring during sleep.^7^

A retrospective cohort study evaluating patients presenting to the ED with non-traumatic headache who underwent magnetic resonance imaging identified age > 40 years and smoking as independent risk factors in multivariate analysis.^8^ Moreover, a secondary subgroup analysis by Chu et al,^9^ which combined data from the HEAD and Headache in Emergency Departments (HEAD)-Columbia studies,^10^,^11^ included 4,489 patients with non-traumatic headache and no neurological findings. This analysis identified age > 50 years, presence of neoplasm, and fever (> 38 °C) as independent predictors of serious intracranial etiologies such as SAH, intracranial hemorrhage, meningitis, hydrocephalus, and vascular dissection. However, in contrast to our findings, the study by Chu et al did not find headache exacerbated by physical activity to be a significant predictor. This discrepancy may be attributed to differences in study populations. Specifically, our study examined a narrower cohort by excluding patients with fever or known intracranial masses.

Subgroup analysis of patients diagnosed with SAH showed that headache aggravated by physical activity is significantly related to SAH, and that syncope is more prevalent in those patients but without a borderline statistical insignificance. However, syncope was also found to be significant in the logistic regression analysis. As generally accepted in the literature, “worst headache ever” and sudden-onset headache does not differ between patients with and without SAH. A recent reanalysis of the HEAD study reported the prevalence of SAH in patients presenting with thunderclap headache as 3.7%, compared to 0.3% in patients without thunderclap headache (*P* = .55).^12^

Contrary to common belief, no single risk factor in a patient’s history exhibits robust diagnostic accuracy for SAH in the ED. A meta-analysis by Carpenter et al investigated the diagnostic accuracy of historical features in patients with suspected SAH. Their findings indicated the following pooled sensitivities and specificities: sudden-onset headache (58% and 50%); loss of consciousness (16% and 95%); vomiting (65% and 72%); and “worst headache of life” (89% and 26%), respectively. However, significant heterogeneity exists across the included studies.^13^

The US Centers for Disease Control and Prevention reported that headache constituted 2.3% of ED visits among female patients and 1.1% among male patients 15–64 years of age; across all age groups, the proportion was 2.7%.^14^ A recent analysis of ED presentations in Türkiye, encompassing more than 5,000,000 patient visits over a period exceeding five years, reported that headache accounted for 4% of all ED presentations among females across all age groups. No male-specific estimate was provided; however, the proportion is likely lower in males and lower when analyses are restricted to adults.^15^ Use of CT for non-traumatic headache varies across countries and is lower in Türkiye (28.9%) than in Europe (46%), Australia/New Zealand (40%), and Hong Kong/Singapore (38%).^16^

Across the six participating centers, approximately 550,000 ED visits occur annually. Assuming headaches account for 3–4% of all presentations, an estimated 24,750 headache visits would be expected over the 18-month study period. Applying an observed ~29% CT use, this corresponds to roughly 7,200 head CT examinations. Notably, the present cohort is a selected subset of non-traumatic headaches particularly focused on a group with greater diagnostic complexity. Therefore, the above calculations might not strictly match to the present cohort.

Beyond overall CT use, non-contrast head CT served as the primary imaging method in our study cohort. Shuaib et al reported only one case with an abnormal contrast-enhanced head CT despite a normal non-contrast head CT among 379 ED patients presenting with non-traumatic headache.^6^ Accordingly, a non-contrast head CT as a first strategy is cost-conscious and may be safer, given the potential adverse effects of iodinated contrast. Consistent with this, non-contrast head CT-first remains common practice across EDs in multiple countries. In a secondary analysis of the HEAD study, Chu et al evaluated CT use for non-traumatic headache with 5,281 patients across Australia, New Zealand, Colombia, France, the United Kingdom, Hong Kong, Singapore, and Türkiye. The imaging modality for head CT in their study included imaging comprised of non-contrast head CT and CT angiography ordered primarily by emergency clinicians,^16^ which is similar to our findings.

## LIMITATIONS

This study had several limitations. A total of 104 (6.8%) patients could not be reached for follow up by telephone at the one-month mark. Consequently, we cannot definitively ascertain whether any intracranial cause was missed within this cohort. Additionally, we intentionally recruited a narrow range of patients to specifically address the diagnostic challenges encountered by emergency physicians in identifying intracranial causes of non-traumatic headaches with no neurological deficits. While this focused approach provides valuable insights into a complex diagnostic area, it may limit the generalizability of the findings to a broader headache population.

The exclusion of febrile patients warrants consideration, given that intracranial infections such as meningitis and encephalitis often present with fever. However, in such cases the clinical presentation typically includes additional neurological findings that necessitate neuroimaging. Furthermore, upper respiratory tract infections are a common cause of ED presentations and frequently involve fever, which generally allows for their distinction from more serious intracranial pathologies.

The study included patients without neurological deficits. However, ensuring consistency in every aspect of the neurological examination is challenging in a multicenter study. In addition, attending physicians tended to be selective in repeating the neurological examination. Finally, despite the relatively large number of study patients, no sample size calculation was performed, which may have resulted in the analyses being underpowered.

## CONCLUSION

In patients presenting to the emergency department with non-traumatic headache and no neurological deficits, headache aggravated by physical activity is a significant indicator for detecting an intracranial cause and subarachnoid hemorrhage alike. Additionally, while age > 50 years is associated with any form of intracranial cause, syncope is specifically linked to subarachnoid hemorrhage within this patient cohort.

## Figures and Tables

**Table 1:** Demographic features and intracranial causes detected in study patients.

Variable	N = 1,522
Age, mean±SD	47.6±16.8
Sex (Male)	643 (42.2)
Sudden onset of pain	762 (50.1)
Alleviated with analgesic	276 (18.1)
History of similar headaches	545 (35.8)
Syncope	92 (6)
Aggravated by physical activity	71 (4.7)
Vomiting	426 (28)
Worst headache ever	901 (59.2)
Intracranial causes^	57 (3.7)
Subarachnoid hemorrhage	20 (35.1)
Subdural hemorrhage	6 (10.5)
Brain mass	5 (8.8)
Ischemic stroke	16 (28.1)
Sinus venous thrombosis	6 (10.5)
Intraparenchymal hemorrhage	2 (3.5)
Meningitis	1 (1.8)
Encephalitis	1 (1.8)

* All data presented as frequencies and rates, unless stated otherwise.

^ Variables determined to be statistically significant.

**Table 2:** Univariate analysis of variables related to patient’s present illness history in the prediction of intracranial pathology.

Variable	Intracranial pathology (+) n = 57	Intracranial pathology (−) N = 1,465	*P*-value
Age > 50 years	39 (68.4)	641 (43.8)	<.001^
Sudden onset	16 (28.1)	746 (51.1)	<.001^
Analgesic response	7 (12.3)	269 (18.4)	.24
Similar headaches in past	19 (33.3)	526 (36.0)	.68
Syncope	6 (10.5)	86 (5.9)	.15
Aggravated by physical activity	7 (12.3)	64 (4.4)	.01^
Vomiting	14 (24.6)	412 (28.1)	.55
Worst headache ever	24 (42.1)	877 (60.0)	<.001^

* All data presented as frequencies and rates, unless stated otherwise.

^ Intracranial causes detected in study patients during the study period.

**Table 3:** Univariate analysis of variables related to the patient’s present illness history in the prediction of subarachnoid hemorrhage.

Variable	Subarachnoid hemorrhage (+) n = 20 (%)	Subarachnoid hemorrhage (−) n = 1,502 (%)	*P*-value
Age > 50 years	10 (50)	670 (44.6)	.63
Sudden onset	9 (45)	753 (50.3)	.64
Analgesic response	2 (10)	274 (18.3)	.56
Similar headaches in past	6 (30)	539 (36)	.58
Syncope	3 (15)	89 (5.9)	.09
Aggravated by physical activity	5 (25)	66 (4.4)	<.001#
Vomiting	2 (10)	424 (28.2)	.07
Worst headache ever	11 (55)	890 (59.4)	.69

* All data presented as frequencies and rates, unless stated otherwise.

# Variables detected to be statistically significant.
